# Exploratory Analyses of Cerebral Gray Matter Volumes After Out-of-Hospital Cardiac Arrest in Good Outcome Survivors

**DOI:** 10.3389/fpsyg.2020.00856

**Published:** 2020-05-06

**Authors:** Aziza Byron-Alhassan, Heather E. Tulloch, Barbara Collins, Bonnie Quinlan, Zhuo Fang, Santanu Chakraborty, Michel Le May, Lloyd Duchesne, Andra M. Smith

**Affiliations:** ^1^Department of Cardiac Prevention and Rehabilitation, University of Ottawa Heart Institute, Ottawa, ON, Canada; ^2^School of Psychology, University of Ottawa, Ottawa, ON, Canada; ^3^Faculty of Medicine, University of Ottawa, Ottawa, ON, Canada; ^4^Cardiology, University of Ottawa Heart Institute, Ottawa, ON, Canada; ^5^Department of Medical Imaging, The Ottawa Hospital, Ottawa, ON, Canada

**Keywords:** out-of-hospital cardiac arrest, gray matter volume, cognition, myocardial infarction, magnetic resonance imaging

## Abstract

**Background:**

Survival rates of cardiac arrest have increased over recent years, however, survivors may still be left with significant morbidity and functional impairment. A primary concern in cardiac arrest survivors is the effect of prolonged hypoxia/ischemia on the brain. The objectives of the present study were threefold: (1) to explore the effect of cardiac arrest on brain gray matter volumes (GMV) in “good outcome” survivors of out-of-hospital cardiac arrest (OHCA), (2) to examine the relationship between GMV, cognitive functioning and arrest factors, and (3) to explore whether OHCA patients differ from a group of patients with myocardial infarction (MI) uncomplicated by cardiac arrest and a group of healthy controls in terms of GMV.

**Methods:**

Medically stable OHCA survivors with preserved neurological function and who were eligible for magnetic resonance imaging scanning (MRI; *n* = 9), were compared to: (1) patients who had experienced a MI (*n* = 19) and (2) healthy controls (*n* = 12). Participants underwent brain MRI on a 3T Siemens Trio MRI scanner and GMV was measured by voxel-based morphometry. A comprehensive neuropsychological assessment was also conducted. Global GMV was compared in the three samples using analyses of variance. The relationships between cognition and GMV were examined within group using correlations.

**Results:**

The OHCA and MI groups showed a similar pattern of differences compared to the healthy control group. Both groups had decreased GMV in the anterior cingulate cortex, bilateral hippocampus, right dorsolateral prefrontal cortex, right putamen, and bilateral cerebellum. There were no significant differences in global or regional GMV between the OHCA and MI groups. Cognitive functioning was correlated with global GMV in the OHCA group; no such correlation was observed in the MI group.

**Conclusion:**

Regional atrophy was observed in OHCA and MI survivors, compared to a healthy control group, suggesting a common mechanism, presumably preexisting cardiovascular disease. Although similar regional volume differences were observed between the MI and OHCA groups, the relationship between GMV and cognition was only observed in OHCA survivors. We suggest the acute hypoxia/ischemia ensuing from the arrest may interact with diminished neural reserve in select brain areas to expose occult cognitive dysfunction.

## Introduction

Survival rates from cardiac arrest are improving (Benjamin et al., 2017) but survivors, even those who have made a seemingly good neurological recovery, are at risk for cognitive impairments that can negatively impact quality of life (Green et al., 2015). During a cardiac arrest, the heart stops beating and blood flow throughout the body and brain ceases until it is restored by either cardiopulmonary resuscitation (CPR) or defibrillation. Without adequate blood flow, the cells of the body and brain are deprived of oxygen and nutrients, and neurons are among the most sensitive cells to this hypoxic/ischemic state. Neuronal death can occur within minutes of a cardiac arrest (Busl and Greer, 2010), and even after successful resuscitation, reintroduction of oxygen to the abnormal biochemical cascades initiated by an arrest can result in reperfusion injuries (Weisfeldt and Becker, 2002). Those most at risk are individuals who experience an out-of-hospital cardiac arrest (OHCA) compared to those who arrest in hospital, as OHCA patients often do not receive immediate medical attention, putting them at particular risk for brain injury or death (Benjamin et al., 2017).

Magnetic resonance imaging (MRI) is often utilized to predict survival in comatose OHCA survivors given its high spatial resolution and ability to detect brain injury *in vivo* (Rossetti et al., 2016; Keijzer et al., 2018). According to the selective vulnerability hypothesis, not all neurons are uniformly affected by hypoxia. Regions of the brain with higher metabolic demands and rich in glutamate receptors are at the greatest risk for brain injury (Busl and Greer, 2010; Björklund et al., 2014). These more vulnerable regions include CA1 cells in the hippocampus, cortical gray matter, and deep gray matter, such as the thalamus and striatum (Busl and Greer, 2010; Gutierrez et al., 2010). Although no definitive prognosticators have been identified, signal abnormalities reflecting cytotoxic edema in regions like the occipital and medial-temporal cortices and the putamen (Wijdicks et al., 2006; Rossetti et al., 2016; Keijzer et al., 2018) are common among those who do not make good neurological recovery. Not only do certain neurons have higher metabolic requirements, some neurons may be at preferential risk due to their location relative to cerebral vascular supply (Torvik, 1984). Cortical and subcortical regions distal to major cerebral arteries are known to be affected in stroke, and also are at increased risk for cell death due to hypoperfusion (Caine and Watson, 2000).

As the majority of neuroimaging research on cardiac arrest survivors to date has focused on prognostication of survival in comatose individuals (Booth et al., 2004; Madl and Holzer, 2004), whether there are similar changes among “good outcome” survivors is still relatively unknown. This is understandable, given that approximately 80% of individuals resuscitated from cardiac arrest do not regain consciousness immediately (Booth et al., 2004). Cognitive deficits, however, are present in 34–50% of cardiac arrest survivors (Moulaert et al., 2009; Green et al., 2015), including those with “good neurological outcome” (Raina et al., 2008; Cronberg et al., 2009; Torgersen et al., 2010; Byron-Alhassan et al., unpublished). Memory dysfunction is the most common area of deficit (Green et al., 2015), though deficits in attention and executive functions are also observed (Harve et al., 2007; Hofgren et al., 2008; Lim et al., 2014; Ørbo et al., 2014). The neurophysiological correlates of these cognitive deficits may be understood using the selective-vulnerability hypothesis. For example, memory dysfunction has been found to correlate with hippocampal reductions in this population (Stamenova et al., 2018; Ørbo et al., 2019). However, a general pattern of widespread volumetric reduction across cortical (Horstmann et al., 2010; Ørbo et al., 2019) and subcortical structures (Horstmann et al., 2010) are commonly observed. As such, a more generalized impact of hypoxic injury across the brain may be the cause of cognitive sequelae, highlighting the importance of examining both regional and whole-brain reductions.

A better understanding of the underlying neuropathological mechanisms may allow better prognostication of which survivors are at greatest risk for persistent cognitive deficit, but few studies have examined neurophysiological sequelae in those who have survived and appear to be doing well neurologically. For example, to our knowledge, only one known study has examined the relationships between cortical thickness, cognitive dysfunction, and two key clinical variables, the duration between arrest and spontaneous circulation (i.e., downtime) and duration of coma after arrest (Ørbo et al., 2019). They found only duration of coma was correlated with cognitive function; subcortical gray matter (GM) regions that, according to the selective vulnerability hypothesis, may also be susceptible to hypoxia were not explored (Busl and Greer, 2010).

Most studies of cognitive dysfunction after cardiac arrest have focused on hypoxic/ischemic mechanism, while the contribution of cardiovascular disease to cognitive dysfunction has been relatively overlooked in OHCA survivors (Cronberg and Lilja, 2015). Cardiovascular disease, a risk factor for cardiac arrest, is well known to be associated with cognitive function (Irani et al., 2009; Jefferson et al., 2011; Cronberg and Lilja, 2015), however, few studies have included a cardiovascular control group when examining the neurophysiological and cognitive outcomes in cardiac arrest survivors (Grubb et al., 2000; Stamenova et al., 2018). In the largest known study of cognitive outcomes after cardiac arrest (Lilja et al., 2015), decreased memory performance was observed not only in the cardiac arrest group but also in the myocardial infarction (MI) group that had not experienced an arrest.

Very few MRI studies have focused specifically on OHCA survivors with relatively good neurological outcome (Grubb et al., 2000; Horstmann et al., 2010; Ørbo et al., 2018, 2019; Stamenova et al., 2018) even though these individuals are known to experience cognitive deficits. To date, research has understandably focused on survivorship, but with novel medical interventions leading to increased survival rates (Chan et al., 2014), a gap has appeared in our understanding of the residual impacts on the brain caused by cardiac arrest. The MRI studies that exist in this area have been limited in scope, perhaps because of the inherent logistic difficulty of sampling patients during such a vulnerable period as following an arrest. Some studies, for example, have included patients who have experienced an arrest due to non-cardiac causes such as electrocution (Horstmann et al., 2010). Others have not included a cardiac control group (Ørbo et al., 2018), or have focused solely on memory functioning when evidence suggests that other cognitive domains are likely to be implicated (Ørbo et al., 2018). To our knowledge, no studies have evaluated individual arrest characteristics in relation to cortical and subcortical brain volumes.

The primary objectives of the present study were: (1) to explore the effect of cardiac arrest on brain gray matter volumes (GMV) in survivors within 3 months of the OHCA; (2) to compare the GMV of OHCA patients to both a group of patients who had experienced an MI uncomplicated by cardiac arrest, and a group of healthy controls; (3) to examine the relationship between GMV and cognitive functioning as measured by a neuropsychological assessment among patients in the OHCA and MI groups; and, (4) to examine the relationships between GMV, cognitive functioning, downtime and duration of coma among OHCA survivors.

## Materials and Methods

### Participants

Participants in the OHCA group were drawn from a larger study of cognitive function in patients with relatively preserved neurological function (Byron-Alhassan et al., unpublished). Participants had to score 1 or 2 on the Cerebral Performance Categories (CPC; Jennett and Bond, 1975), indicating normal, mild or moderate impairments in cerebral functioning but independence in activities of daily living. In an effort to distinguish cognitive dysfunction caused by cardiac arrest from any pre-existing cognitive disturbance related to underlying cardiovascular or cerebrovascular disease, patients who had been hospitalized for MI without cardiac arrest were enrolled to serve as a control group. MI participants were recruited either while in hospital post-MI or at an outpatient follow-up appointment. Healthy control (HC) participants were recruited by word of mouth at the hospital and university, or nomination by patients or study team members.

Exclusion criteria for all groups included pre-existing conditions that could influence performance on neuropsychological testing such as current substance abuse disorder, history of traumatic brain injury with loss of consciousness >30 min, and serious pre-existing neurological or psychiatric illnesses (e.g., stroke or schizophrenia). Participants were also excluded if they were non-MRI compatible (e.g., had an implanted cardioverter defibrillator), claustrophobic, or if they had any physical or mental health problems that precluded them from lying in relative stillness for the duration of the scans. Participants were also excluded if they were not fluent in English or younger than 18 years of age. Participants received a $25 gift card for participating in the study. This study was approved by the Ottawa Health Science Network Research Ethics Board. Informed, written consent was obtained from all participants.

### Procedures

Participants were either scanned while still in hospital or shortly after hospital discharge by a medical radiation technologist. A clinical neuroradiologist (SC) reviewed all MRI scans for any clinically significant abnormalities. Any patients with acute or gross structural abnormalities (i.e., infarcts, tumors) that would influence group-level comparisons were excluded from final analyses.

### Measures

#### Demographic Information and Arrest Factors

Participants reported their age, sex, ethnicity, education level, employment status, and smoking history in an interview with research personnel. Where possible, this information was verified by chart review. Downtime and length of coma (i.e., time in days from arrest to successful extubation, when coma persisted after initial return-of-spontaneous circulation) were obtained from patient’s medical charts and emergency medical services records.

#### Cognitive Assessment

Cognitive functioning was measured using the Neuropsychological Assessment Battery (NAB), a paper-pencil neuropsychological assessment. comprised of five modules measuring memory, attention, language, spatial, and executive functions, respectively. A score is generated for each module and these are summed to obtain the global NAB index, a global measure of cognitive performance (Stern and White, 2003). The global NAB index reflects individual performance compared to the standardization sample in standard scores (e.g., mean value = 100, standard deviation = 15), where higher values equate to better performance. The NAB provides normative data stratified by age, education, and sex from a large standardization sample comprised of neurologically healthy individuals. Given the extensive normative data that exists for this battery, cognitive functioning was not assessed in the HC group. Psychometric properties of the NAB, including reliability and validity, have been well established and it has been shown to be sensitive to cognitive impairment in a variety of clinical populations (Stern and White, 2003).

#### Magnetic Resonance Imaging

Imaging data were collected on a 3.0 Tesla Siemens TRIO MR scanner. Participants lay flat on an automated bed fitted with a 32-channel head coil. They were in two-way communication at all times with the medical radiation technologist performing the scanning. A 3D FLASH (TR/TE 11.2/21 ms, flip angle 60°, field of view (FOV) 26 × 26 cm^2^, 256 × 256 matrix, slice thickness 1.5 mm) anatomic image was acquired for structural analyses.

### Data Analyses

#### Sample Characteristics

Patient demographics (i.e., age, sex, and medical comorbidities), cognitive performance (i.e., mean NAB scores), and time since event (i.e., time to cognitive testing and time to scanning) were compared between groups using analysis of variance (ANOVA) and t-tests for continuous variables, and chi-squared tests for categorical variables.

#### Voxel-Based Morphometry

Structural images were analyzed using the SPM12 toolbox with the DARTEL algorithm^1^. Before analyses, all images were reoriented to be aligned with the AC-PC line. We followed the step-by-step processing sequence suggested by the DARTEL toolbox for the voxel-based morphometry (VBM) analysis: (1) The MR images were first field bias-corrected to correct non-uniform fields. (2) Next, using the tissue probability maps based on the International Consortium of Brain Mapping (ICBM), the images were segmented to GM, white matter (WM), and cerebrospinal fluid (CSF). (3) The average study-specific GM and WM templates were subsequently computed and created. (4) After an initial affine registration of the GM DARTEL templates to the tissue probability maps in Montreal Neurological Institute (MNI), non-linear warping of individual GM images was performed to the DARTEL GM template and an individual flow field for each participant was created. (5) The individual GM images were normalized into the MNI space with a 1.5 mm × 1.5 mm × 1.5 mm voxel size with the normalized images modulated to ensure the relative volumes of GM were preserved following the spatial normalization procedure. (6) The modulated, normalized GM images were then smoothed with an 8 mm FWHM Gaussian kernel. (7) Finally, the tissue volumes utility of SPM12 was used to extract the average global (whole-brain) value of GM (GMV) for each participant based on the segmentation files, which was applied in the further analyses.

### Group-Level Analyses

#### GMV Between-Group Differences

The smoothed, normalized individual GM images were applied in the second-level group analyses using SPM12. The voxel-wise ANOVA analyses and *post hoc* two-sample *t*-tests were conducted to examine the GMV differences among three groups (i.e., HC vs. OHCA, HC vs. MI, OHCA vs. MI). Individual age and global GMV were included in the SPM group-level GLM model and controlled for as covariates of no interests in all imaging analyses. The global GMV was included as a nuisance variable to control the effect of individual differences in the global GMV. Reported regions were derived at the threshold of uncorrected *p* < 0.001, cluster size >30 voxels at the whole-brain level. Multiple comparison corrections were then applied to all regions at the cluster level to verify the results. Both family-wise-error (FWE) and small volume corrections (SVC) were performed (Poldrack et al., 2008). For SVC correction, anatomical independent regions of interests (ROIs) were defined as masks using the WFU pickatlas AAL template^2^, then the SVC function in SPM for the correction was applied. All regions were reported regardless of the significance after multiple comparison corrections.

#### Correlation Between GMV and Cognitive Functioning

Next, ROI analyses were performed to explore the relationship between cognitive functioning and the regional GMV, as well as the global GMV. Regional GMV that differed significantly for the patient groups compared with the HC group were extracted using marsbar^3^ based on the voxel-wise analyses results. Partial correlation analyses were then conducted between the cognitive performance scores and the regional GMV with global GMV and age as covariates of no interests. Also, the partial correlation analysis was performed between the cognitive functioning and global GMV, with age as a covariate of no interest using IBM SPSS 25.0.

#### Cardiac Arrest Clinical Variables and GMV Regression Analyses

To further explore the relationship between downtime and duration of coma with GMV among the OHCA sample, whole-brain spatial regression analysis was conducted in SPM12. Clinical values (i.e., downtime, days of coma) were entered in the model as covariates to examine which regional GMVs were correlated with clinical values. Threshold applied was uncorrected *p* < 0.005, cluster size >30 voxels at the whole-brain level.

## Results

### Sample Characteristics

A description of the sample and clinical variables can be found in Table 1. Participants across the three groups ranged in age from 30 to 84 years (*M*_*age*_ = 59.65, SD_*age*_ = 11.61). There were no group differences in age [*F*(2,37) = 1.699, *p* = 0.197] or sex [*n*_*female_OHCA*_ = 2, *n*_*female_MI*_ = 2, *n*_*female_HC*_ = 3, χ^2^ (2, *N* = 40) = 1.502, *p* = 0.560]. Patients in the MI group generally had higher rates of cardiovascular risk factors (e.g., diabetes, dyslipidemia, and hypertension) however differences were not statistically significant (see Table 1). Participants in the OHCA group completed cognitive assessment and MRI scanning closer to the date of their cardiac event than the MI group. However, time to test and time to scan were not significant covariates in the between-group analyses of cognitive functioning [*F*(1,24) = 3.972, *p* = 0.058], or global GMV [*F*(1,24) = 0.064, *p* = 0.802], suggesting that they did not contribute to group differences in cognitive functioning or global GMV.

**TABLE 1:** Demographics, cognitive performance, and clinical variables.

	OHCA	MI	HC	*p*-Value
*N*	9	19	12	
Mean age in years (SD)	59.89 (14.63)	62.58 (9.69)	54.83 (11.32)	0.197
Sex (% male)	77.8	89.5	75	0.560
Hypertension (%)	3 (33.33)	12 (63.16)	n/a	0.228
History of MI (%)	2 (22.22)	3 (15.79)	n/a	1.000
Dyslipidemia (%)	2 (22.22)	12 (63.16)	n/a	0.103
Diabetes (%)	1 (11.11)	4 (21.06)	n/a	0.645
Mean NAB Index (SD)	101.33 (17.68)	108.42 (11.30)	n/a	0.294
Mean downtime (minutes; SD)	10.78 (7.03)	n/a	n/a	n/a
Mean duration of coma (days; SD)	3.11 (4.51)	n/a	n/a	n/a
Mean time to test (SD)	13.78 (13.04)	40.52 (21.96)	n/a	0.001
Mean time to scan (SD)	26.67 (16.21)	62.84 (28.63)	n/a	0.001

*NAB, Neuropsychological Assessment Battery; time to test refers to time in days from index event to date of cognitive testing; time to scan refers to time in days from index event to date of magnetic resonance imaging scans; OHCA, out-of-hospital cardiac arrest; MI, myocardial infarction; HC, healthy controls; SD, standard deviation; n/a, not applicable.*

### Cerebral GMV

**FIGURE 1:**
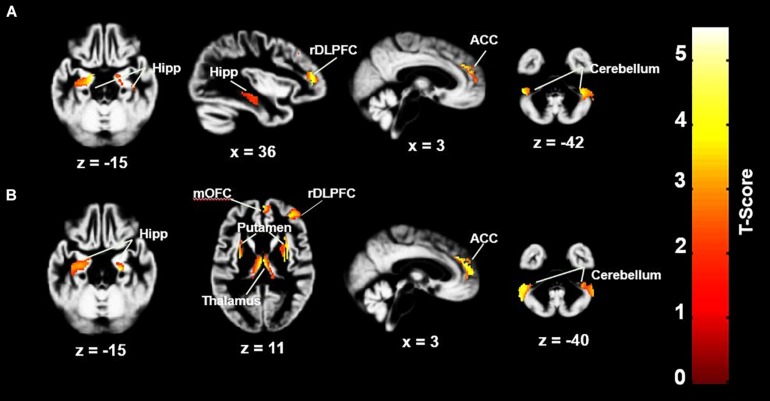
GMV differences between HC group and MI and OHCA group. **(A)** HC > OHCA; **(B)** HC > MI. ACC, anterior cingulate cortex; rDLPFC, right dorsolateral prefrontal cortex; Hipp, hippocampus; mOFC, medial orbitofrontal cortex. Statistical inferences were performed at a threshold of uncorrected *p* < 0.001 at the whole-brain level and *p* < 0.05, family wise error or small volume corrected at the cluster level. There were no significant differences in GMV between the OHCA and MI groups at the whole-brain level.

Group mean values for global GMV, did not differ significantly between the three groups. Although this study focused on GMVs, global WM volumes were also compared, and did not differ significantly between the three groups (Table 1), [*F*(6,68) = 1.818, *p* = 0.109, partial η^2^ = 0.138]. In whole-brain analyses controlling for age and global GMV, between-group comparisons revealed regional GMV decreases in the both the OHCA and MI groups in comparison to the HC group; the OHCA group had decreased GMV in the anterior cingulate cortex (ACC), right dorsolateral prefrontal cortex (rDLPFC), bilateral hippocampus, right putamen, and bilateral cerebellum areas; the MI group had decreased GMV in the ACC, medial orbitofrontal cortex (OFC), rDLPFC, bilateral hippocampus, thalamus, bilateral putamen, and bilateral cerebellum areas. Most of these regions remain significant after the SVC correction at the cluster level (*p* < 0.05) (see Figure 1 and Table 2) or were approaching significance. There were no significant differences in GMV between the OHCA and MI groups at the whole-brain level even before correction. Further exploratory analyses conducted without defining the cluster size limitation revealed an additional small cluster (six voxels) of reduced GM in the lDLPFC for the MI group compared to the HC group. These results are presented in Table 2.

**TABLE 2:** GMV differences between HC and MI, OHCA.

	MNI coordinates			Peak	Cluster-level correction (*p*-value)	
			
Brain regions	*x*	*y*	*z*	*t*-score	FWE	SVC
**HC > OHCA**						
ACC	2	36	29	4.16	0.967	0.090
rDLPFC	36	42	14	4.07	0.641	0.048
rHipp	15	−9	−12	4.17	0.870	0.019
lHipp	−14	−9	−11	4.96	0.552	0.016
rPutamen	32	−2	12	3.68	0.937	0.062
rCerebellum_Crus2	44	−38	−47	4.80	0.414	0.051
lCerebellum_7b	−47	−44	−50	3.97	0.294	0.020
**HC > MI**						
ACC	2	45	20	4.79	0.151	0.016
mOFC	9	42	−14	4.74	0.020	0.004
rDLPFC	38	45	5	4.55	0.339	0.022
lDLPFC	−38	33	12	3.55	0.929	0.253
rHipp	17	−9	−12	3.61	0.796	0.011
lHipp	−14	−9	−11	3.98	0.777	0.022
Thalamus	−2	−18	12	4.51	0.384	0.024
rPutamen	29	−6	12	3.92	0.019	0.006
lPutamen	−29	−9	12	4.19	0.648	0.050
rCerebellum_Crus2	44	−38	−47	5.38	0.135	0.028
lCerebellum_Crus1	−56	−62	−36	4.59	0.006	0.002

*Table presents three-dimensional Montreal Neurological Institute (MNI) coordinates for significant brain regions with peak cluster *t*-score value and corrected significance values. r, right side; l, left side; ACC, anterior cingulate cortex; mOFC: medial orbitofrontal cortex; DLPFC: dorsolateral prefrontal cortex; Hipp, Hippocampus; FWE, family-wise error correction; SVC, small volume correction; OHCA, out-of-hospital cardiac arrest; MI, myocardial infarction; HC, healthy controls.*

### Cerebral GMV and Cognitive Functioning

Mean cognitive performance in both groups was within the average range as compared to the normative data and did not differ between the cardiac groups in this sample [*t*(26) = −1.101, *p* = 0.294; for an in-depth analysis of cognitive performance between OHCA and MI groups, refer to Byron-Alhassan et al., unpublished). Regional volumes that differed significantly for the patient groups compared with the HC group were extracted (i.e., ACC, OFC, DLPFC, hippocampus, thalamus, putamen, and cerebellum) and correlated with cognitive functioning in each cardiac group. Although no regional volumes were correlated with cognitive performance in the MI or OHCA group when controlling for age and global GMV, global GMV alone was correlated with cognitive performance in the OHCA group but not the MI group (see Table 3). A strong positive correlation between GMV and cognitive performance was observed. However, a *z*-test comparing the correlation between GMV and total NAB scores in the OHCA group to the MI group only approached statistical significance (*z* = −1.62, *p*_*one–tailed*_ = 0.053).

**TABLE 3:** Correlations between GMV and cognitive performance on the global NAB Index.

Brain regions	MI	OHCA
Total GMV	0.308	0.798*
ACC	0.279	0.396
mOFC	–0.050	0.348
rDLPFC	0.339	0.044
bHipp	0.045	0.574
Thalamus	0.33	0.663
bPutamen	–0.302	0.326
bCerebellum	0.233	–0.403

Cognitive function is measured with the Neuropsychological Assessment Battery, scores are standardized to a normative sample for age, gender, and education. Correlations are controlled for age and total GMV. r, right side; l, left side; b, bilateral; ACC, anterior cingulate cortex; mOFC, medial orbitofrontal cortex; DLPFC: dorsolateral prefrontal cortex; Hipp, Hippocampus; OHCA, out-of-hospital cardiac arrest; MI, myocardial infarction. *p < 0.05.

### Cerebral GMV and Clinical Variables

**FIGURE 2:**
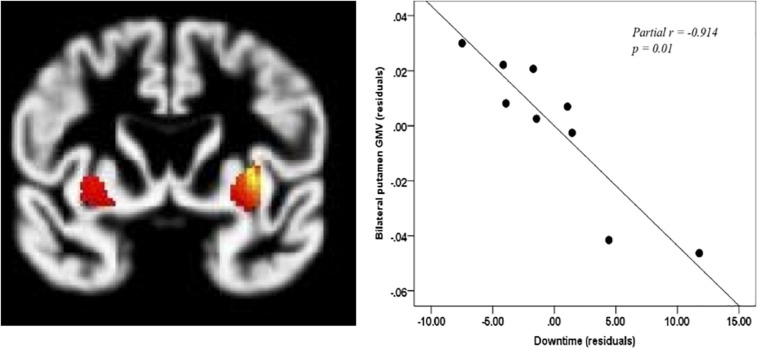
Correlation between downtime and putamen. Coordinates of bilateral putamen: left: –30, 5, 3; right: 32, 3, 6, *p*_*corr*_ < 0.005. Only right putamen remains significant after small volume correction *p* < 0.05.

Downtime ranged from 1 to 21 min in the OHCA group (*M* = 10.78, SD = 7.03). When controlling for age, global GMV did not correlate significantly with downtime (*R*^2^ = −0.568, *p* = 0.142). However, when controlling for age and global GMV, downtime was negatively correlated with GMV in the left putamen (−30, 5, 3, *p* < 0.005) and right putamen (32, 3, 6, *p* < 0.005) although, only the correlation with right putamen retained significance after SVC (see Figure 2).

Days of coma ranged from 0 to 14 (*M* = 3.11, SD = 4.51) with four patients not having experienced any coma. When controlling for age, global GMV did not correlate significantly with days of coma (*R*^2^ = −437, *p* = 0.292). At the whole-brain level, when controlling for age and global GMV, no regional GMVs correlated significantly with days of coma.

## Discussion

The purpose of this study was to evaluate GMV in a sample of OHCA survivors, who had made seemingly good neurological recovery, as compared to MI and HC groups. These participants were drawn from a comprehensive study of cognitive functioning after OHCA, which allowed us to explore the association between cognitive functioning and GMV. This study is novel in its inclusion of both an MI control group and a healthy control group. Their inclusion allowed us to further address the issue of the etiology of cognitive dysfunction after cardiac arrest, particularly the relative contributions of pre-existing cardiovascular disease and acute hypoxia/ischemia.

The findings of this study revealed cortical and subcortical volumetric reductions in select brain regions of OHCA survivors. These regions included the ACC, rDLPFC, bilateral hippocampus, right putamen, and bilateral cerebellum. Reductions in hippocampal volumes were expected in this population given reports that memory impairment is the predominant cognitive sequela of OHCA (Green et al., 2015; Moulaert et al., 2009) and the known vulnerability of the hippocampus to hypoxia (Busl and Greer, 2010; Björklund et al., 2014). The ACC and right dorsolateral prefrontal cortex are both key structures in executive functioning (Cummings, 1993), another commonly observed domain of cognitive impairment for survivors of OHCA (Green et al., 2015). Though volumetric reductions in these regions fit the cognitive profile for OHCA survivors, we also observed similar reductions in the MI group. These results suggest that pathology in these regions may result from risk factors common to both cardiac groups. Some of the regions with decreased volume observed in this study have been linked with cardiovascular risk factors (O’Donnell et al., 2016). For example, decreased prefrontal cortex volumes have been observed in patients with hypertension (Raz et al., 2003) and activations in the anterior cingulate, which purportedly play a role in the autonomic nervous system, has been found to correlate with behavioral stress (Gianaros et al., 2005). Reduced cerebellar volumes have been observed in heart failure patients and may relate to decreased perfusion (Alosco and Hayes, 2015). Our findings suggest an increased need for studies of the pathophysiologic relationships between cardiac functioning and the brain.

In this study, we did not observe any differences between the OHCA group and either control group in terms of global GMV. Ørbo et al. (2018) found decreased global GMV compared to healthy controls among individuals who were comatose upon arrival in hospital after OHCA, but not among those who were conscious upon arrival. This may be pertinent to our results, given that approximately half (*n* = 4) of our sample were non-comatose hospital admissions. While we suspect that GMV is likely reduced in the OHCA population, our analyses were likely underpowered to observe such differences. The fact that our participants were scanned closer to the index event than in other studies may further account for some differences in findings.

Only the OHCA group had significant correlations between cognitive functioning and volumetric data; lower global GMV volume was correlated with worse cognitive performance. This correlation was not observed in the MI group, although the strength of the two correlations did not differ significantly. We would expect, with a larger sample, that the MI group would show a correlation between GMV and cognition, as has been observed in the literature (Kanai and Rees, 2011). However, we observed a strong correlation within our small sample of OHCA survivors, suggesting that volume loss among OHCA patients may be related to increased risk for cognitive impairment. Such correlations between brain volumes and cognitive function have been observed in other MRI studies of cardiac arrest survivors. Grubb et al. (2000) similarly found that, although volumes were reduced in some regions, only total brain volumes were significantly correlated with cognitive performance and Stamenova et al. (2018) likewise found that correlations between cognitive functioning and hippocampal volumes were only apparent in their OHCA group, and not within the MI group. One possible explanation could be that pre-existing cerebrovascular disease burden diminishes neural reserve rendering an individual more vulnerable to the effects of cerebral hypoxia.

The OHCA group performed worse on cognitive testing in all domains than the MI group and, although these differences were not statistically significantly different in this sample. The lack of difference between the OHCA group and MI group may be a result of a small sample size, or due to sampling bias in the OHCA given that individuals who are eligible for MRI cannot have an implanted cardioverter defibrillator, a commonly used preventative measure after OHCA. To investigate this possibility, we compared participants in the present study to the larger sample of OHCA who underwent cognitive assessment from which they were drawn (*n*_*OHCA*_ = 77). Results showed that those who participated in the imaging sub-study performed significantly better on cognitive testing than those who did not (*M*_*MRI*_ = 101.33 SD = 17.68, M_*COG_ONLY*_ = 83.48, SD = 14.42, *U* = 471.50, *p* = 0.009), although this was not the case for the MI group (*M*_*MRI*_ = 108.42, SD = 11.30, *M*_*COG_ONLY*_ = 104.16, SD = 12.93, *U* = 560.50, *p* = 0.194). These results indicate that individuals who participated in our MRI study performed significantly better on cognitive testing than those who participated in a study involving cognitive assessment alone. Despite this, we found reduced GMV in this group of OHCA survivors who were doing relatively well from a cognitive standpoint, which may suggest a large effect in the greater population. With increasing survival rates and the advent of MRI-compatible defibrillators, future studies may see larger sample sizes to further understand this interesting population.

Downtime was correlated with volumetric data in order to understand the potential relationship between the duration of hypoxia and cerebral GMV. Unlike other studies which have focused primarily on the hippocampus as a region that is primarily susceptible to hypoxia (Ørbo et al., 2018; Stamenova et al., 2018), our results revealed a strong, negative correlation between the putamen and downtime (partial *r* = −0.914, *p* = 0.01). The putamen has been identified as a region that is susceptible to cytotoxic edema in individuals who do not have a favorable outcome from coma after cardiac arrest (Rossetti et al., 2016; Keijzer et al., 2018). The putamen is a key region in motor control and it also contributes to cognitive processes such as working memory (Arsalidou et al., 2013). Days of coma was not correlated with any regional volumes at the whole-brain level. Although another study identified significant correlations between days of coma, cortical thickness, and some aspects of cognition (Ørbo et al., 2019), the sample differed from the one in this study in that it included only patients comatose upon hospital arrival and therefore likely experienced more severe neurological impairment.

This study has some limitations. Survival rates of cardiac arrest are low, and many patients are treated with implanted cardioverter-defibrillators, which are not MRI-compatible; thus, the individuals included in MRI studies may not be representative of typical cardiac arrest survivors. As previously mentioned, compared to our larger study of cognitive functioning, those who participated in this neuroimaging sub-study demonstrated better cognitive performance. Eligibility restrictions also contributed to a small sample size; this is a common limitation of imaging studies of cardiac arrest survivors (*n* = 9–13; Horstmann et al., 2010; Stamenova et al., 2018; Ørbo et al., 2019). Uneven sample sizes were also a limitation given that OHCA survivors were more difficult to recruit than the control groups. We wished to evaluate patients prior to hospital discharge at the point in time when rehabilitation planning typically occurs; however, medical complications prevented many patients from participating within this time frame which may have further detracted from the representativeness of the sample. We have found in our larger study that the MI patients perform somewhat better on the NAB than the American standardization sample, suggesting that the test norms may not be ideal for a Canadian sample. As a result, inferences about cognitive functioning in both groups as compared to the healthy control group, and its relation to volumetric data, may be distorted. Finally, hypoxic-ischemic brain injury and reperfusion injuries are dynamic processes that change over time (Busl and Greer, 2010). The current data were obtained at a time when neurological recovery was still ongoing. Identifying neural patterns in the acute phase which may predict long-term functioning are important next steps for future research.

## Conclusion

Our results highlight the importance of considering baseline neurophysiology when evaluating the neural impact of OHCA. Findings suggest that there may be an interplay of acute and chronic neural changes, such that acute hypoxia/ischemia from the arrest may give rise to subtle deficits in cognitive functions subserved by regions with diminished neural reserve due to pre-existing cerebrovascular disease. Future imaging studies that include a functional component will be important in understanding the functional implication of volumetric reductions observed in this population.

## Data Availability Statement

The datasets presented in this article are not readily available because all requests for data must be reviewed by our institution and/or ethical review board. Requests to access the datasets should be directed to the corresponding author.

## Ethics Statement

The studies involving human participants were reviewed and approved by the Ottawa Health Science Network Research Ethics Board. The patients/participants provided their written informed consent to participate in this study.

## Author Contributions

HT, AS, BC, BQ, SC, ML, and LD conceived of the study and designed the analyses. AB-A collected the data with the support of BQ, SC, ML, LD, and AS. AB-A and ZF contributed to and performed data analysis. AB-A wrote the manuscript with the support of HT, BC, ZF, and AS. AB-A, HT, BC, BQ, ZF, SC, ML, LD, and AS critically reviewed and accepted the contents of the manuscript.

## Conflict of Interest

The authors declare that the research was conducted in the absence of any commercial or financial relationships that could be construed as a potential conflict of interest.

## Footnotes

1 https://www.fil.ion.ucl.ac.uk/spm/

2 https://www.nitrc.org/projects/wfu_pickatlas/

3 http://marsbar.sourceforge.net/
